# Uncovering the factors that affect earthquake insurance uptake using supervised machine learning

**DOI:** 10.1038/s41598-023-48568-6

**Published:** 2023-12-03

**Authors:** John N. Ng’ombe, Kwabena Nyarko Addai, Agness Mzyece, Joohun Han, Omphile Temoso

**Affiliations:** 1 Department of Agribusiness, Applied Economics and Agriscience Education, North Carolina A&T State University, Greensboro, NC 27411 USA; 2 Department of Accounting, Finance and Economics, Griffith Business School, Griffith University, Nathan, QLD 4111 Australia; 3 Department of Economics, Agriculture and Social Sciences, California State University, Stanislaus, Turlock, CA 95382 USA; 4 Department of Agricultural Economics and Agribusiness, University of Arkansas, Fayetteville, AR 72701 USA; 5 UNE Business School, University of New England, Armidale, NSW 2351 Australia

**Keywords:** Environmental social sciences, Natural hazards

## Abstract

The escalating threat of natural disasters to public safety worldwide underlines the crucial role of effective environmental risk management tools, such as insurance. This is particularly evident in the case of earthquakes that occurred in Oklahoma between 2011 and 2020, which were linked to wastewater injection, underscoring the need for earthquake insurance. In this regard, from a survey of 812 respondents in Oklahoma, USA, we used supervised machine learning techniques (i.e., logit, ridge, least absolute shrinkage and selection operator (LASSO), decision tree, and random forest classifiers) to identify the factors that influence earthquake insurance uptake and to predict individuals who would acquire earthquake insurance. Our findings reveal that influential factors that affect earthquake insurance uptake include demographic factors such as older age, male gender, race, and ethnicity. These were found to significantly influence the decision to purchase earthquake insurance. Additionally, individuals residing in rental properties were less likely to purchase earthquake insurance, while longer residency in Oklahoma had a positive influence. Past experience of earthquakes was also found to positively influence the decision to purchase earthquake insurance. Both decision trees and random forests demonstrated good predictive capabilities for identifying earthquake insurance uptake. Notably, random forests exhibited higher precision and robustness, emerging as an encouraging choice for earthquake insurance modeling and other classification problems. Empirically, we highlight the importance of insurance as an environmental risk management tool and emphasize the need for awareness and education on earthquake insurance as well as the use of supervised machine learning algorithms for classification problems.

## Introduction

The current surge in the occurrence, intensity, and severity of natural disasters (e.g., hurricanes/cyclones, earthquakes, floods, and others) around the world has called for the need for various insurance packages as a risk management tool to guarantee the safety of nations and their citizenry^1–3^. However, the extent to which people see and understand the consequences of natural disasters as well as the degree to which the disaster threatens their safety are rarely considered in earthquake insurance design. Survey research has suggested that the ability to comprehend the consequences of natural hazards in any location is linked to several demographic variables of respondents (including age, gender, and race) as well as a variety of local contextual factors (e.g., length of residence in the state, risk perception, government aid, and others)^4–6^.

We conjecture that these factors have played a significant role in numerous global contexts where earthquakes or other natural disasters have occurred. According to Pynn and Ljung ^7^, the primary motivation for purchasing disaster insurance among North Dakotans is their perception of risk. Furthermore, Botzen and Bergh^8^ conducted a contingent valuation survey in the Netherlands to elicit individual risk beliefs and flood insurance, and found that individuals are willing to pay for disaster insurance based on their perception of flood risk. In the Oklahoma context in particular, Li et al.^9^ found that perception on the efficacy of protective action (e.g., expected outcome of insurance) has a significant role in risk mitigation action for tornados and earthquakes.

A study by Seifert et al.^10^ that compared flood insurance in Germany and the Netherlands found that people are inclined to insure for natural disasters such as floods based on their risk experience, risk perception, and the charity hazard. Niyibizi et al.^11^ found that, on average, Oklahomans prefer lower earthquake risks and support regulations on activities linked to human-induced earthquakes in their state to reduce seismic activity. In addition, Abbas et al.^12^ found that in Pakistan, factors such as age, non-agricultural income, and preconceptions about the effectiveness of insurance can influence an individual’s willingness to pay for flood insurance, while Oral et al.^13^ discovered that in Turkey, earthquake preparedness is influenced not only by an individual's experience with earthquakes but also by cultural factors. Moreover, Greer et al.^14^ and Choi et al.^15^ argue that in the U.S., political ideology could significantly affect decision-makers’ perceptions of hazards, but its effects vary by the type of hazard.

Taking the foregoing expositions together, insurance uptake is important because natural disasters can cause significant economic, environmental, and financial losses, impacting not only individuals but also entire communities, regions, and countries. The costs associated with natural disasters can vary greatly depending on their scale and severity, but they can include a wide range of expenses such as damage to infrastructure, homes, and businesses; environmental degradation; loss of income; loss of life and reduced economic growth^16,17^. An example is Hurricane Katrina, one of the most catastrophic hurricanes in recorded history, which caused damages exceeding US$100 billion^2,18^, of which only $62 billion worth of the damaged property was insured^19^.

Another example is the devastating earthquake that struck Sichuan Province in China on May 12, 2008, causing over 88,670 deaths, over 374,000 injuries, and the destruction of an estimated 5.5 million structures^20^. Wang et al.^21^ observed that in response to the repeated earthquakes, the Chinese government launched the Catastrophe Insurance Pilot Program in Chuxiong in 2014. This program aimed to transfer the individual risk of injury and property loss to the insurance market, thus reducing the burden on the government and cutting down on expenditures^20^. Indirect costs, such as disruptions to supply chains and transportation networks, lost productivity, and increased insurance premiums, can also be significant. In some cases, the economic costs of natural disasters and earthquakes can be so substantial that they can destabilize national economies, particularly in developing countries with limited resources and infrastructure to cope with such events^22^. Consequently, it is crucial to develop strategies and policies for managing and mitigating the immediate and long-term effects of natural disasters and earthquakes.

Moreover, despite the potentially enormous cost of the hazards, each individual has a different attitude toward risk preparedness action and hazard control policy depending on their perspective on hazards. For instance, Choi and Wehde^23^ and Murphy et al.^24^ explain that individuals’ perception on hazard and corresponding policy significantly affect not only their risk preparedness action but also their level of compliance toward hazard control policy. Moreover, Wehde and Choi^25^ argue that individual’s perception and trust in hazard control policy may vary depending on the type of hazard. Therefore, without accounting for potential behavioral factors on risk preparedness decision for a given disaster (e.g., purchasing insurance for earthquake), it would be difficult to design an effective policy. Against the foregoing, this study has two objectives. The first is to analyze the factors that influence the decision to purchase earthquake damage insurance. The second objective is to predict which individuals are more likely to obtain earthquake insurance in Oklahoma, given the state's increased vulnerability to earthquakes from 2010 to 2020^26,27^. To realize these twin objectives, we use a variety of supervised machine learning techniques, encompassing logistic regression, ridge regression, and LASSO, to identify the influential factors of earthquake insurance uptake. In addition, decision trees and random forest algorithms are utilized for predictive modeling. The primary goal of the research is to provide insights on how to design policies aimed at encouraging more individuals and businesses to obtain earthquake damage insurance, and minimizing the risk of severe damage and financial loss in the event of an earthquake. The results of this study will provide policymakers with insights on how to design and evaluate feasible methods of implementing earthquake insurance as a compensation mechanism. The findings may also help insurance companies develop more effective marketing strategies to encourage more individuals and businesses to purchase earthquake insurance. Individuals and businesses can also use the study's findings to inform their decision-making process regarding earthquake insurance purchase, potentially resulting in lower financial losses in the event of an earthquake. According to Franco^28^, insurance can be a powerful ‘ex-ante’ strategy in an earthquake mitigation framework when combined with other approaches, because it can help achieve other important goals such as safe building construction and land development regulations.

The state of Oklahoma is a compelling case study for this research due to the significant increase in earthquakes associated with fracking between 2010 and 2020^11,26,27,29–31^. While the state experienced less than two M3.0 earthquakes per year on average from 1978 to 2008, the Oklahoma Geological Survey^32^ reported a sharp increase in earthquakes from 41 to 903 between 2010 and 2015, all which exceed M3.0 on the Richter scale. Although scientific research has provided strong evidence linking fracking to an increase in the number of earthquakes in Oklahoma, the state's risk management and protective action options against induced earthquakes have raised concerns among all individuals and businesses. The legal system's insufficient compensation for earthquake victims, as reported by Konschnick^33^ and Ng'ombe and Boyer^34^, adds to the uncertainty. Furthermore, even though most earthquakes recorded in Oklahoma between 2011 and 2016 were relatively small, some caused significant damage to homes and businesses^33^. For instance, the 5.7 magnitude earthquake that occurred in Prague, Oklahoma in 2011 caused injury to two people and destroyed 14 homes and is believed to be linked to wastewater injection^34,35^. These events underscore the importance of studying the determinants of earthquake damage insurance uptake in Oklahoma to mitigate the financial and physical damages caused by future earthquakes.

The paper makes the following contributions to existing literature. First, by identifying the factors that influence earthquake insurance uptake, this study provides empirical evidence that helps explain the decision-making process of individuals and businesses regarding earthquake insurance uptake. In doing so, the supervised machine learning algorithms used, which include logit, ridge regression, and least absolute shrinkage selection operator (LASSO), are useful in that such models, ridge regression and LASSO in particular, can identify influential factors through variable selection. When applied in this context, ridge regression and LASSO help in selecting a subset of covariates (predictor variables) that have the most influence on earthquake insurance uptake. The LASSO can act as a feature selection method, keeping only the most relevant factors influencing the decision to acquire earthquake insurance while shrinking the coefficients of less importance to zero^36,37^. Our study's findings also shed light on why some individuals and businesses are hesitant to purchase earthquake insurance, despite the increased risk of earthquakes in some regions. This is important because potential earthquake risks in Oklahoma are expected to remain for some time^38^, and this may not be particular to Oklahoma, but many other world regions are susceptible to earthquakes.

Second, we contribute to the earthquake prediction policy problem by identifying attributes that predict individuals who can purchase earthquake insurance based on covariates, by using robust supervised machine learning models that include decision trees and random forests. Earthquake insurance uptake may involve non-linear relationships and complex interactions among factors. Machine learning algorithms like decision trees and random forests are particularly adept at capturing such complexities, which can be critical for understanding the nuanced influences on insurance uptake^39,40^. When compared to traditional econometric approaches, machine learning models are more suitable for prediction problems because they can improve the accuracy and efficiency of uncovering the determinants of earthquake insurance uptake^36,37^. Using decision tree and random forest algorithms for prediction allows us to uncover potential complex relationships between the features and the target variable in addition to important variables that predict earthquake insurance uptake.

## Methods and materials

### Data sources

Our data was collected from a 2017 earthquake survey that was conducted online in Oklahoma using Qualtrics. The data is part of the research by Ng’ombe and Boyer^34^ which established how much of the earthquake damage the oil and gas industry should be liable for, in terms of what earthquake victims endure. This study focuses on the factors associated with the decision by Oklahoma residents to purchase home/property insurance against earthquakes in the state. The data were collected from the U.S. state of Oklahoma and its counties that experienced a rise in earthquakes linked to fracking. To administer the survey, it was first approved by the Oklahoma State Institutional Review Board (IRB), and then conducted through Survey Sampling International (SSI), a reputable organization that employs numerous methods to recruit respondents. The survey was conducted with 1,153 individuals through SSI, of which 813 of them successfully completed the survey. According to Ng’ombe and Boyer^34^, SSI has panels of potential respondents that respond to online surveys for a given fee. A series of questions on people’s attitudes toward earthquakes in the state and their demographic, and socio-economic characteristics were asked in the survey. The variables used in this study are described in Table 1. Columns 1 and 2 respectively include variable names and their definitions. The response variable is earthquake insurance. It represents people who responded Yes or No to a question that asked them whether they insured their residence or property against earthquake-related damage.

**Table 1:** Variable definitions.

Name of the variable	Variable description
Response variable	
Earthquake insurance uptake	= 1 if the respondent has his/her residence/property insured against earthquake damage, 0 o/w
Explanatory variables	
Socio-demographics	
Age	Age of the respondent (years)
Gender	Gender of the respondent (= 1 if male, 0 o/w)
Ethnicity: Hispanic	= 1 if Yes for being Hispanic, Latino or of Spanish origin, 0 o/w
Race: White	= 1 if Yes for being White, 0 o/w
Race: African American or Black	= 1 if Yes for being Black or African-American, 0 o/w
Race: American Indian	= 1 if Yes for being American Indian, 0 o/w
Race: Asian	= 1 if Yes for being Asian, 0 o/w
Belongs to two or more races	= 1 if Yes for belonging to two or more races, 0 o/w
Democrat	= 1 if respondent identified as a Democrat, 0 o/w
Republican	= 1 if respondent identified as a Republican, 0 o/w
Independent	= 1 if respondent identified as belonging to the Independent party, 0 o/w
Years lived in Oklahoma	The period in years the respondent has spent living in Oklahoma
Rents the property	= 1 if the respondent’s residence is rented, 0 o/w
Education: less than high school	= 1 if the respondent’s maximum education level is less than high school, 0 o/w
Education: high school	= 1 if the respondent’s maximum education level is high school, 0 o/w
Education: associate degree	= 1 if the respondent’s maximum education level is an Associate degree, 0 o/w
Education: undergraduate degree	= 1 if the respondent’s maximum education level is an undergraduate degree, 0 o/w
Education: graduate degree	= 1 if the respondent’s maximum education level is a Master’s degree, 0 otherwise
Education: professional degree	= 1 if the respondent’s maximum education level is a Professional degree e.g. MBA, 0 o/w
Incomeless$25,000	= 1 if the respondent’s income/year is less than $25,000, 0 o/w
Inc$25000_$49,999	= 1 if the respondent’s income/year is from $25,000 to $49,999, 0 o/w
Inc$50000_$74,999	= 1 if the respondent’s income/year is from $50,000 to $74,999, 0 o/w
Inc$75000_$99,999	= 1 if the respondent’s income/year is from $75,000 to $99,999, 0 o/w
Inc$10000_$124,999	= 1 if the respondent’s income/year is from $100,000 to $124,999, 0 o/w
Socio-demographics	
Inc$125000_above	= 1 if the respondent’s income/year is upwards of $125,000, 0 o/w
Respondents’ attitudes toward earthquakes in Oklahoma	
No earthquake-related damage	= 1 if the house/property not damaged by earthquakes from the year 2011, 0 o/w
Earthquake-related damage is minor	= 1 if the house/property’s had earthquake-related cracks since 2011, 0 o/w
Earthquake-related damage is moderate	= 1 if the house/property’s walls or brickwork had earthquake-related damage by falling since the year 2011, 0 o/w
Earthquake-related damage is major	= 1 if the house/property’s foundation or rockwork masonry had earthquake-related damage since the year 2011, 0 o/w
Other earthquake-related damage	= 1 if the house/property had earthquake-related damage of other form since the year 2011, 0 o/w
Abrupt stoppage of wastewater injection	= 1 if the respondent wants wastewater injection should be stopped abruptly, 0 o/w
Believes wastewater injection be stopped following science	= 1 if the respondent wants wastewater injection should be abolished following scientific findings, 0 o/w
Believes the main source of revenue to the state are the oil and gas companies	= 1 if the respondent believes that the oil and gas industry is the major source of revenue for the state, 0 o/w
Believes oil and gas companies are the main source of jobs in the state	= 1 if the respondent believes oil and gas industry is the major source of jobs in the state, 0 o/w
Earthquake-related safety concern	= 1 if the respondent is concerned about his/her safety from earthquakes, 0 otherwise
State should regulate environmental quality	= 1 if the respondent believes that the state government should regulate environmental quality, 0 o/w
Oil and gas companies at residential property	The number of oil and gas companies operating on respondent’s property
Works in the oil and gas industry	= 1 if the relative or respondent works in the oil and gas industry, 0 o/w
Oil and gas leases revenue	= 1 if the relative or respondent obtains revenue from O&G leases,0 o/w
Expects to work in oil and gas companies one day	= 1 if the respondent expects to be hired in the oil and gas industry one day, 0 o/w
Has experienced earthquakes in the past	= 1 if the respondent has experienced earthquakes in the past, 0 o/w
Wastewater injection is responsible for earthquakes in Oklahoma	= 1 if the respondent believes wastewater injection is responsible for earthquakes in Oklahoma, 0 o/w

Independent variables are classified into two sets. The first set of independent variables comprises socio-demographics such as age, gender, and race of the survey respondents. The second set comprises a range of variables relating to respondents’ attitudes toward earthquakes in Oklahoma. Table 2 shows descriptive statistics for these variables based on to the entire sample, with and without earthquake insurance. The *t*-test statistics for the mean differences of the variables between the two groups of respondents are presented in the last column of Table 2. The total sample size is 812 with 14.4% (114 respondents) reporting that their property/residence was insured against earthquake damage. In terms of the mean differences of variables by insurance uptake status, we can see that the sample was not purely homogenous in their responses as most of the variable means were statistically different from zero between the two groups. For example, the mean age difference between people with and without insurance was 1.01 years, which is statistically different from zero – those with insurance were older. A similar observation can be made for the numerous variables considered.

**Table 2:** Descriptive statistics of selected variables.

Variable name	Full sample (N = 812)		With insurance (N = 114)		Without insurance (N = 698)		*t* tests
	Mean	SD	Mean	SD	Mean	SD	
Respondent has an earthquake insurance	0.144	0.347					
Socio-demographics							
Age	40.507	15.909	41.377	16.931	40.365	15.732	1.011***
Gender	0.260	0.439	0.307	0.461	0.252	0.434	0.055***
Ethnicity: Hispanic	0.054	0.226	0.070	0.256	0.052	0.221	0.019***
Race: White	0.820	0.384	0.807	0.395	0.822	0.382	-0.015*
Race: African American or Black	0.036	0.186	0.018	0.131	0.039	0.193	-0.021***
Race: American Indian	0.081	0.273	0.070	0.256	0.083	0.276	-0.013**
With insurance (N = 114)Race: Asian	0.017	0.130	0.026	0.160	0.016	0.125	0.010***
Belongs to two or more races	0.036	0.186	0.053	0.223	0.033	0.179	0.019***
Democrat	0.289	0.454	0.219	0.414	0.301	0.459	-0.082***
Republican	0.372	0.483	0.482	0.500	0.354	0.478	0.129***
Independent	0.339	0.473	0.298	0.458	0.345	0.475	-0.047***
Years lived in Oklahoma	8.246	9.735	10.895	10.287	7.814	9.573	3.081***
Rents the property	0.499	0.500	0.263	0.440	0.537	0.499	-0.274***
Education: less than high school	0.097	0.296	0.053	0.223	0.105	0.306	-0.052***
Education: high school	0.413	0.492	0.281	0.449	0.434	0.496	-0.153***
Education: associate degree	0.195	0.396	0.246	0.431	0.186	0.389	0.059***
Education: undergraduate degree	0.202	0.401	0.298	0.458	0.186	0.389	0.112***
Education: graduate degree	0.094	0.291	0.123	0.328	0.089	0.285	0.034***
Education: professional degree	0.262	0.440	0.122	0.327	0.285	0.451	-0.162***
Incomeless$25,000	0.294	0.456	0.289	0.454	0.295	0.456	-0.006
Inc$25000_$49,999	0.219	0.414	0.307	0.461	0.205	0.404	0.102***
Inc$50000_$74,999	0.116	0.320	0.123	0.328	0.115	0.319	0.008
Inc$75000_$99,999	0.062	0.240	0.096	0.295	0.056	0.230	0.041***
Inc$10000_$124,999	0.047	0.211	0.061	0.240	0.044	0.206	0.017***
Inc$125000_above	.048	0.211	0.096	0.007	0.056	0.002	0.041***
Respondents’ attitudes towards earthquakes in Oklahoma							
No earthquake-related damage	0.773	0.419	0.773	0.419	0.782	0.413	-0.063***
Earthquake-related damage is minor	0.167	0.373	0.193	0.395	0.163	0.370	0.029***
Earthquake-related damage is moderate	0.026	0.159	0.061	0.240	0.020	0.140	0.041***
Earthquake-related damage is major	0.026	0.159	0.018	0.131	0.027	0.163	-0.010***
Other earthquake-related damage	0.007	0.086	0.009	0.093	0.007	0.084	0.002
Respondents’ attitudes toward earthquakes in Oklahoma							
Abrupt stoppage of wastewater injection	0.361	0.480	0.360	0.480	0.361	0.480	-0.001
Believes wastewater injection be stopped following science	0.590	0.492	0.579	0.494	0.592	0.492	-0.013
Believes main source of revenue to OK is the oil and gas industry	0.682	0.466	0.649	0.477	0.688	0.463	-0.039***
Believes main source of jobs to OK is the oil and gas industry	0.776	0.417	0.789	0.408	0.774	0.418	0.016
Earthquake-related safety concern	0.591	0.492	0.623	0.485	0.586	0.493	0.037***
State should regulate environmental quality	0.772	0.419	0.789	0.408	0.769	0.421	0.020***
Oil and gas companies at residential property	0.193	0.644	0.421	1.184	0.156	0.493	0.265***
Works in the oil and gas industry	0.167	0.373	0.263	0.440	0.152	0.359	0.111***
Oil and gas leases revenue	0.148	0.355	0.246	0.431	0.132	0.338	0.114***
Expects to work in oil and gas companies one day	0.062	0.240	0.132	0.338	0.050	0.218	0.081***
Has experienced earthquakes in the past	0.755	0.430	0.781	0.414	0.751	0.432	0.030***
Wastewater injection is responsible for earthquakes in Oklahoma	0.570	0.495	0.605	0.489	0.564	0.496	0.041***

### Independent variables

The variables included in all our models are shown in Table 1. They are based on a review of literature on people’s attitudes toward earthquakes [e.g., 11, 25, 34, 36, 42–52]. We included socio-demographic variables because they are expected to influence people’s decision to have earthquake insurance as well as to help predict which individuals would acquire earthquake insurance. in accordance with existing literature, we anticipate that female respondents are more likely than males to own earthquake insurance because females are warier of environmental threats and calamities^11,41^. Regarding demographic factors such as race, ethnicity, education, and income, we anticipate a heterogeneous relationship with the likelihood of having earthquake insurance. For example, Ansolabehere and Konisky^42^ found that minority groups are more skeptical and concerned about coal and natural gas plants located near their homes, which we believe would motivate them to purchase earthquake damage insurance. In a separate study, Boudet et al.^43^ observed that politically conformist respondents had at least an undergraduate degree, and older respondents were more likely to support hydraulic fracturing in the United States. Also, Ng’ombe and Boyer^34^ found varying levels of earthquake-related liability levels that respondents would assign to fracking firms in Oklahoma for any damage, which suggests that similar varying outcomes could be expected regarding their decision to purchase earthquake insurance.

Moreover, we expect respondents who have lived in Oklahoma for a longer period to more likely own earthquake insurance because they would have more previous experience with earthquakes, especially since 2009, when the state started to experience numerous earthquakes^10^. As in Ng’ombe and Boyer^34^, we anticipate that renters will be less likely to have earthquake damage insurance than property owners. By political affiliation, we anticipate that Democrats (Republicans) are more (less) likely to have earthquake insurance because they have shown greater (lesser) willingness to accept fracking related benefits for regulation^11^. Boudet et al.^43^ and Davis and Fisk^44^ observed that Democrats are more committed to controlling wastewater injection in order to prevent potential earthquakes than Republicans.

Following Ng’ombe and Boyer^34^, we included the extent of earthquake damage respondents’ residence or property incurred in the past when assessing respondents’ attitudes toward earthquakes in Oklahoma. These include minor damage, moderate damage, major damage, and other damage as defined in Table 1. There have been many lawsuits in Oklahoma by residents whose property incurred damage from earthquakes, but success stories related to compensation are few^45,46^. However, we expect that people whose property or house incurred any earthquake-related damage will prefer to have their property insured against earthquakes, especially since most insurers have stated that they would be able to insure against both natural and man-made earthquakes in Oklahoma^47^.

Other earthquake-related factors that we expect to influence the decision to have earthquake insurance include people’s concerns about earthquake damage, the state’s obligation to regulate wastewater injection, earthquake experience, beliefs, and knowledge of the importance of oil and gas companies to Oklahoma. For example, we hypothesize that those who want wastewater injection to be abruptly stopped or who prefer to have earthquake insurance are warier of the risks of earthquakes to their property and therefore more likely to purchase earthquake insurance.

### Supervised machine learning algorithms

To achieve its objectives, this study uses the following supervised machine learning (ML) algorithms: logit model, ridge regression, LASSO, decision trees, and random forest. We chose supervised ML over alternative methods because it served our research as a classification problem. With supervised ML, the algorithm is trained to learn the mapping between the input data (predictor variables) and the output data (dependent variable), so that it can select the influential variables of the output data and depending on the model used, also make predictions on new, previously unseen data.

Supervised machine learning is the method of choice for applications like ours, where a specific target variable must be both explained by influential input factors and predicted. In our case, we employ supervised ML to unravel the variables that shape individuals' decisions regarding earthquake insurance acquisition while also predicting which respondents are likely to make a purchase. To clarify, supervised ML effectively categorizes variables into two crucial categories: output (e.g., the decision to purchase insurance or not) and input (e.g., respondent characteristics like risk perception). This categorization assumes a significant correlation and, potentially, a causal relationship between the labeled input and output variables, offering a robust framework for our analytical endeavors^40^. On the other hand, in unsupervised ML, the algorithm is given a dataset with no pre-existing labels or outputs (i.e., no correlation/causation assumptions between variables). The algorithm then attempts to find patterns or structure in the data on its own, without any guidance or supervision. Unsupervised learning is used in applications where the researcher is interested in discovering hidden patterns or groupings in the data, such as clustering similar customers together for targeted marketing^39,48^.

### Logit, ridge regression, and LASSO

Let the dependent variable ${y}_{i}$ follow a Bernoulli distribution. In this context, ${y}_{i}$ represents whether an individual has their residence or property insured against damage from earthquakes. The probability of detecting whether an individual has their residence or property insured against damage from earthquakes and $P({y}_{i}=1)$ denotes the probability of detecting insurance coverage based on the available predictors in the data. This way, we have a binary classification model that explains the probability of classes ${y}_{i}=1$ or ${y}_{i}=0$ using the predictors described in Sect. 'Methods and materials'. Following James et al.^40^, the logit classifier is

1 $$P\left( {y_{i} = 1} \right) = e^{{x_{i} \beta }} /1 + e^{{x_{i} \beta }} ,$$

where observations in the database are represented by *i*, whereby *i* = 1*, …, N*, and $\beta$ corresponds to unknown parameters to be estimated, ${x}_{i}$ is a vector of explanatory variables. Maximum likelihood estimation of the following log-likelihood function allows us to estimate the parameter estimates

2 $${\mathcal{L}}\left( \beta \right) = \mathop \sum \limits_{i = 1}^{N} \left( {y_{i} x_{i} \beta - {\text{log}}\left( {1 + e^{{x_{i} \beta }} } \right)} \right)$$

In high-dimensional settings, where the number of predictors is large, collinearity between the predictors can lead to unstable estimates and unreliable predictions. In such cases, penalized regression models are commonly employed to improve the accuracy and interpretability of the model. Penalized regression techniques, such as ridge regression and LASSO, add a constraint to the equation to regularize the model^39^, resulting in reduced coefficients. This, in turn, shrinks the coefficients of less influential variables towards zero, improving the general performance of the model^40^. In this study, we applied the ridge classifier and LASSO, which impose a penalty on the logit model to avoid overfitting and identify influential variables in the decision to acquire earthquake insurance.

The ridge classifier adds a fine-tuning parameter $\lambda \ge 0$ to Eq. (2). Estimation of the coefficients using the ridge classifier is achieved by maximizing the following modified version of Eq. (2)

3 $${\mathcal{L}}_{ridge} \left( \beta \right) = \mathop \sum \limits_{i = 1}^{N} \left( {y_{i} x_{i} \beta - {\text{log}}(1 + e^{{x_{i} \beta }} } \right)) - \lambda \mathop \sum \limits_{j = 1}^{k} \beta_{j}^{2} ,$$

where *k* is the total number of penalized coefficients. However, the ridge classifier includes all the coefficients in the final model by adding a squared magnitude of the coefficient $\beta$ as a penalty term. Therefore, if $\lambda \to \infty$*,* there would be overfitting^37,48^. Increasing $\lambda$ reduces the variance but raises the bias resulting in the model being less accurate and more precise. We therefore used cross-validation when selecting the optimal value of $\lambda$ to minimize the validation error.

The LASSO offers an alternative regularization procedure in which a number of inputs is potentially eliminated from the model, thereby bypassing limitations of a ridge classifier. Introduced by Hastie et al.^48^, the LASSO’s log-likelihood to be maximized is

4 $${\mathcal{L}}_{LASSO} \left( \beta \right) = \mathop \sum \limits_{i = 1}^{N} \left( {y_{i} x_{i} \beta - {\text{log}}(1 + e^{{x_{i} \beta }} } \right)) - \lambda \mathop \sum \limits_{j = 1}^{k} \left| {\beta_{j} } \right|.$$

The LASSO algorithm adds a penalized term to the traditional logit model which sets the coefficients of less influential variables to zero. It therefore selects only those inputs that are most relevant among the potentially many variables in the databases. Cross-validation was also used to select the $\lambda .$

### Decision trees

Decision trees are a powerful and widely used tool for solving classification problems in machine learning. A decision tree is a tree-like model that uses a set of input features to make predictions about the target variable^39,49^. The use of a decision tree in this study is appealing because it provides sufficient visual information to predict which individuals are likely to insure against earthquake damage or not. The decision tree model consists of nodes and branches, where each node represents a feature, and each branch represents a possible outcome for that feature. The tree is constructed by recursively splitting the data into subsets based on the values of the input features until the subsets become homogeneous with respect to the target variable^48^.

Regarding training the model, the process involves searching for the best features to split the data and creating a hierarchy of nodes and branches that best separates the data into different classes. In our case, this produces a tree structure that can be used to predict earthquake damage insurance uptake for the new and previously unseen data. One of the most important benefits of decision trees is their interpretability. Unlike other machine learning algorithms that can be difficult to interpret, such as random forest (next section), decision trees are simple to understand and can provide valuable insights into the decision-making process. Decision trees can also handle non-linear relationships between the input features and the target variable, making them a valuable tool in many real-world applications^39^. While decision trees maybe easier to interpret, they can suffer from overfitting, where the model becomes too complex and captures noise in the data. To address this issue, several techniques have been developed, including pruning and ensemble methods like random forests^40^.

### Random forests

Another ML technique we use to achieve our second objective is a random forest classifier. A random forest classifier has a wide range of applications, including image classification, natural language processing, and fraud detection^50^. First introduced by Breiman^51^, random forest classifiers are an ensemble learning method that combines the outputs of multiple decision trees to make a final prediction. During the training phase, the algorithm constructs several decision trees on different subsets of the training data. At each split in a decision tree, a random subset of the input features is selected as candidates for the split. This process helps to reduce overfitting and improve the generalization ability of the model. The final prediction is made by aggregating the predictions of all individual decision trees^40^. The use of random forests has become increasingly popular due to their ability to handle high-dimensional data and capture complex nonlinear relationships in the data, making them an effective tool for many real-world applications.

Random forests have several advantages over other classification algorithms. According to Kassambara^39^ and James et al.^40^, random forests are robust to overfitting, can handle large datasets with high dimensionality, can capture complex nonlinear relationships between input features and output labels, and are relatively easy to use and require minimal parameter tuning. In the present study, let *x* be a vector of inputs described in Table 1. The inputs in Table 1 will help us to predict *y*, that reveals whether an individual has his/her property insured against earthquake damage. By so doing, the training procedure for random forests applies bootstrap aggregation or bagging to tree learners (see James et al.^40^ and Breiman^51^ for more details). Thus, for a training set $x={x}_{1}, \dots ,{x}_{n}$ with responses $y={y}_{1}, \dots ,{y}_{n}$, bootstrap aggregation continually selects a random sample (*K* times) with replacement of the training data sets to fit trees to the following samples.

For *b* = 1, …, *B*:

- Sample $n$ training samples with replacement from $x, y$, and let these be called ${x}_{b},{y}_{b}.$
- Train a classification tree ${k}_{b}$ on ${x}_{b},{y}_{b}.$
- Upon completion of the training, make predictions for unseen samples ${x}{\prime}$ by taking the majority vote in the case of classification trees or by averaging the predictions made from all the individual regression trees on ${x}{\prime}$ in the case of non-classification trees as follows
  5 $$\hat{k} = \frac{1}{B}\mathop \sum \limits_{b = 1}^{B} k_{b} (x{\prime} ),$$

Such a bootstrapping algorithm is considered to improve model performance because it decreases model variance without increasing the bias. Therefore, an estimate of the uncertainty of prediction is the standard deviation of the prediction from all individual regression trees *x’*^40^.

6 $$\sigma = \sqrt {\frac{{\mathop \sum \nolimits_{b = 1}^{B} (k_{b} (x{\prime} ) - \hat{k})^{2} }}{B - 1}} .$$

The optimal number of trees, B, can be determined through two methods: cross-validation or the out-of-bag error (OOBE), as described in James et al.^40^. The OOBE measures the average prediction error for each training sample ${x}_{i}$ using the trees that did not include ${x}_{i}$^37^. In general, the testing and training errors tend to level off after a certain number of trees have been fitted. As noted by Silveira et al. ^37^, this is the basic bagging technique for trees. However, random forests differ from this pattern in one keyway: they use a modified learning algorithm that selects a random subset of features at each candidate split during the learning process. Silveira et al.^37^ contend that the selection of highly predictive features can lead to correlated results in the *B* trees, which can affect the accuracy of the model for earthquake damage insurance. However, using a subset of features in each split, typically the square root of the total number of features, can help to prevent correlation and improve accuracy^40,49^.

### Performance measures

The performance of logistic regression, ridge regression, and LASSO models was assessed by examining the magnitude and signs of the coefficients. Ridge regression and LASSO, being regularization techniques, tend to push coefficients towards zero. Consequently, variables retaining non-zero coefficients in LASSO or having relatively larger coefficients in ridge regression are considered more influential^39^. This evaluation provided insights into the extent to which these models effectively identify the most influential factors affecting earthquake insurance uptake.

As previously mentioned, in the case of decision trees and random forests, individuals were classified as having a high likelihood of having earthquake damage insurance if their predicted probability exceeded 0.5. Therefore, to evaluate the performance of the decision tree and random forest models for predictive modeling, various performance measures were employed, including accuracy, sensitivity, specificity, and precision which are computed as:

7 $$Accuracy = \frac{TN + TP}{{TN + TP + FN + FP}}$$

8 $$Precision = \frac{TP}{{TP + FP}}$$

9 $$Sensitivity = \frac{TP}{{TP + FN}}$$

10 $$Specificity = \frac{TN}{{TN + FP}}$$

where *TN* are true negatives, *FN* are false negatives, *TP* are true positives, and *FP* are false positives. True negatives refer to when the model identifies a data point as part of the negative class, and this identification is correct (e.g., the model identifies an individual A as non-buyer of insurance through learning, and it does match with an actual decision of A in the data). In the same context, *FN*, *TP*, and *FP* happen by false negative (e.g., identify A as a non-buyer but does not match with actual decision), true positive (e.g., identify A as a buyer and does match with actual decision), and false positive (e.g., identify A as a buyer but does not match with actual decision), respectively. These metrics collectively provided insights into the decision trees and random forest models' effectiveness in correctly identifying those likely to purchase earthquake insurance.

Moreover, each performance measure falls within the range of 0 to 1, with higher values indicating better model performance for each metric. For instance, if *FN* and *FP* are unlikely to happen (i.e., the model accurately identifies individuals’ positive/negative decisions through learning), the denominator of *Accuracy* measure will be closer to the numerator. Therefore, the measure will be closer to 1. *Accuracy* depicts a fraction of cases correctly classified out of the total cases. *Precision*, also known as positive predicted value, measures the proportion of correctly predicted positive cases out of the total number of positive cases in the dataset^39^. *Sensitivity* – or recall or the true positive rate, calculates the proportion of positive cases that are correctly classified out of the total number of real positive cases in the dataset^40^. Finally, *Specificity* measures the proportion of negative cases that are correctly identified out of the total number of negative cases in the dataset. These performance measures are typically derived from a confusion matrix, which is a 2 × 2 table that summarizes the results of a machine learning algorithm by classifying observations as *TP*, *TN*, *FP*, and *FN*.

### Estimation strategies

**Figure 1:**
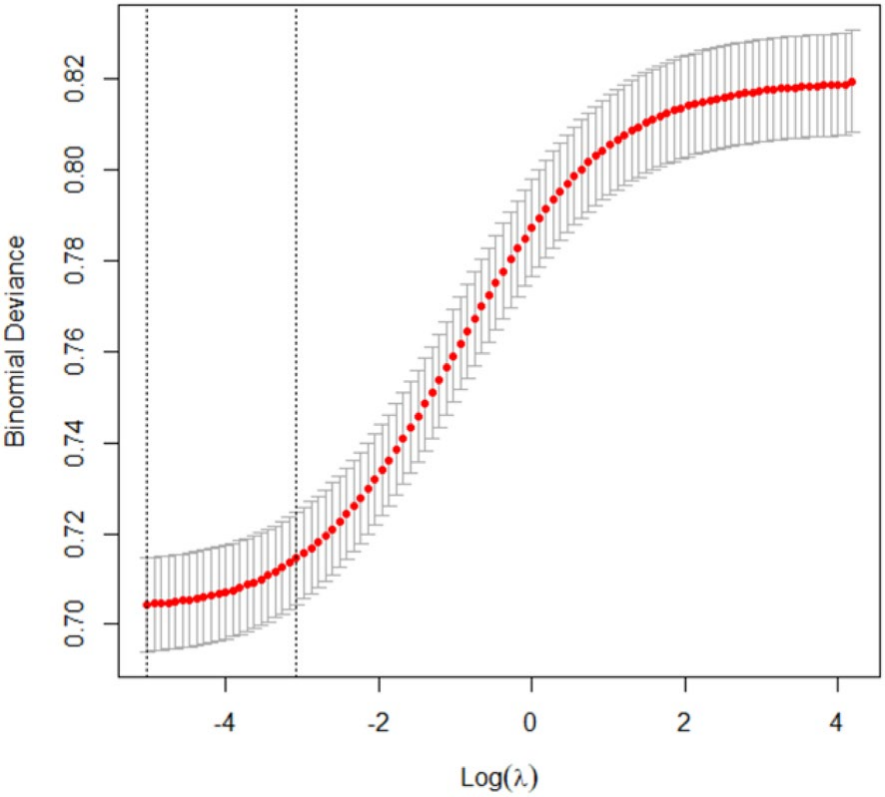
Plot of cross validation error for the ridge classifier.

**Figure 2:**
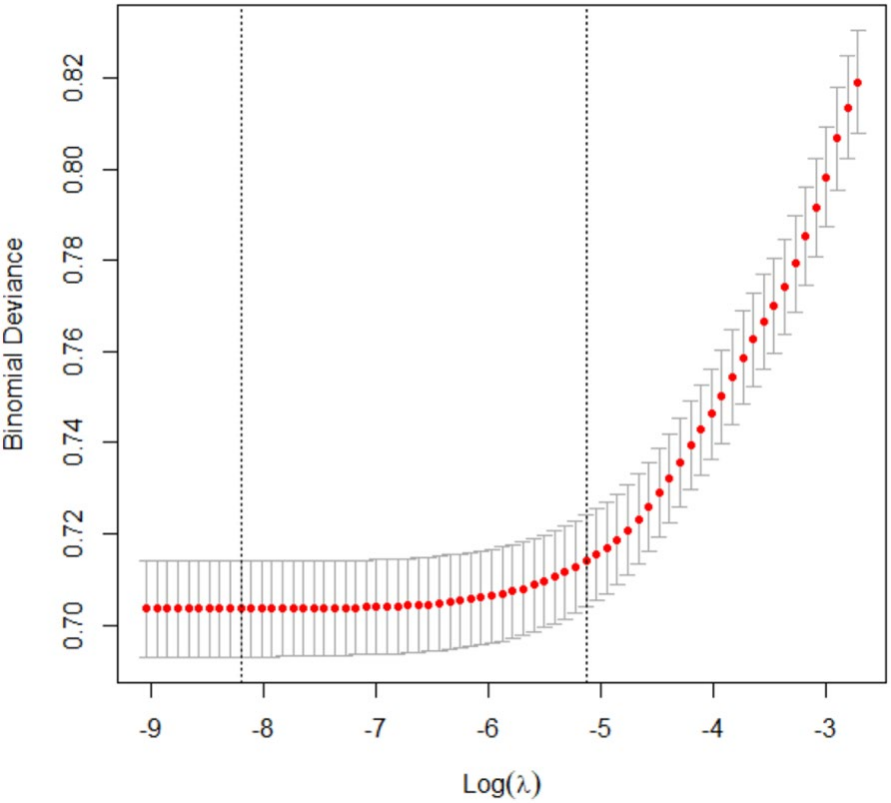
Plot of cross validation error for the LASSO classifier.

In this study, we utilized a range of R software packages including *glmnet*, *caret*, *rpart*, and *rattle* to estimate all our models^52–56^. To obtain reliable estimates, we divided our data into separate training and testing sets^40^. The training set comprised 70% of the total observations and was used to estimate model parameters, while the remaining 30% were used for out-of-sample estimation and prediction in the testing set. We used the *glmnet* package to implement ridge and LASSO classifiers and determine the optimal λ values that minimized cross-validation prediction error. Specifically, we performed cross-validation to identify the optimal values of $\lambda$ that gave the best models and found that $\lambda$ = 0.0066 and $\lambda$ = 0.0003 were optimal for ridge regression and LASSO, respectively^40^. We generated plots of the cross-validation error as a function of log ($\lambda$) for the ridge and LASSO classifiers (Fig. 1 and 2, respectively). Note that increasing the value of $\lambda$ for the ridge regression (LASSO) tends to shrink coefficients of factors that affect earthquake insurance toward zero (or exactly zero).

As for predictive models, particularly the decision tree classifier, due to the large number of variables used to predict individuals who insure their property against earthquake, the decision tree generated was too large, necessitating pruning. Pruning involves simplifying the model by removing branches or nodes that do not contribute to its predictive power and prevents overfitting. We pruned the decision tree using recursive partitioning and selected the complexity parameter using tenfold cross-validation^55^. As mentioned before, to further improve on the performance of the decision tree model, we also employed random forests. Random forests can reduce the model's variance and improve its accuracy^40^.

## Results

### Factors affecting earthquake insurance uptake

Table 3 presents results from logit, ridge regression, and LASSO. We find that the LASSO model did not eliminate any coefficients, probably due to the small value of the shrinkage parameter chosen by cross-validation. Therefore, we present the results of all three classifiers collectively in Table 3. Moreover, regularization approaches such as ridge and LASSO tend to shrink the coefficients towards zero, which can lead to biased estimates of the standard errors. Consequently, the *glmnet* package used to estimate these models does not provide standard errors for the coefficients^39,52^. While the absence of standard errors limits the interpretability of the coefficients, we note that the magnitudes and signs of the coefficients are consistent across the three models. This also confirms that the results in Table 3 are robust. Given this consistency and the significance of the coefficients in the logit model, we focus our analysis on the significant coefficients of the results identified by this model.

**Table 3:** Estimation results from logit, ridge and LASSO classifiers.

Model name	Logit classifier		Ridge classifier	LASSO
Variable name	Estimates	Std. error	Estimates	Estimates
Dep. variable: Respondent has an earthquake insurance				
Socio-demographics				
Age	−0.037***	0.010	−0.008	−0.024
Square of age	0.000***	0.000	0.000	0.000
Gender of respondent	0.173***	0.059	0.186	0.186
Ethnicity: Hispanic	0.289**	0.114	0.410	0.422
Race: African American or Black	−0.113	0.186	−0.115	−0.087
Race: American Indian	−0.077	0.103	−0.051	−0.041
Race: Asian	0.850***	0.171	0.854	0.922
Belongs to two or more races	0.463***	0.142	0.330	0.319
Democrat	−0.795***	0.078	−0.721	−0.844
Republican	0.012	0.068	0.070	0.004
Years lived in Oklahoma	0.016***	0.003	0.021	0.021
Rents the property	-1.061***	0.068	−0.952	−1.039
Education: high school	0.098	0.117	0.077	0.006
Education: associate degree	0.682***	0.120	0.416	0.536
Education: undergraduate degree	0.859***	0.119	0.609	0.727
Education: graduate degree	0.724***	0.135	0.557	0.689
Inc$25000_$49,999	0.769***	0.087	0.686	0.837
Inc$50000_$74,999	0.917***	0.091	0.763	0.936
Inc$75000_$99,999	0.219*	0.113	0.146	0.271
Inc$100000_$124,999	0.555***	0.124	0.326	0.454
Inc$125000_above	0.519***	0.135	0.375	0.510
Respondents’ attitudes toward earthquakes in Oklahoma				
Earthquake-related damage is minor	−0.035	0.069	−0.086	−0.092
Earthquake-related damage is moderate	0.929***	0.132	0.860	0.883
Earthquake-related damage is major	−0.221	0.187	−0.381	−0.354
Other earthquake-related damage	0.582**	0.282	0.503	0.579
Abrupt stoppage of wastewater injection	−0.124	0.127	−0.029	−0.013
Believes wastewater injection be stopped following science	−0.014	0.121	0.078	0.105
Source of revenue to Oklahoma is the oil and gas industry	−0.662	0.073	−0.559	−0.625
Source of jobs in Oklahoma is the oil and gas industry	0.367	0.084	0.281	0.330
Dep. Variable: Respondent has an earthquake insurance				
Respondents’ attitudes toward earthquakes in Oklahoma				
Earthquake-related safety concern	0.371***	0.059	0.253	0.281
State should regulate environmental quality	0.127*	0.071	0.086	0.090
Oil and gas companies at residential property	0.433***	0.038	0.481	0.507
Works in the oil and gas industry	0.416***	0.073	0.401	0.416
Revenue from oil and gas leases	0.431***	0.071	0.396	0.396
Expects to work in oil and gas companies one day	0.402***	0.101	0.498	0.493
Has experienced earthquakes in the past	0.009	0.066	0.036	0.013
Wastewater injection is responsible for earthquakes in Oklahoma	0.014	0.062	0.024	0.007
Intercept	−2.140***	0.289		
Model fit diagnostics				
Akaike Information Criterion	7,199			
Deviance	7,120		7,139	7,121
Lambda			0.0066	0.0003

***, **, *Statistical significance at 1%, 5% and 10% level.

The findings in Table 3 reveal that several socio-demographic factors have a significant impact on earthquake insurance uptake. These factors include age, gender, ethnicity, race, and political affiliation. Interestingly, Democrats were found to be less likely to have earthquake insurance compared to Independents. Moreover, the length of time a person has lived in Oklahoma was also found to influence their likelihood to insure against earthquakes. Other factors that were found to affect the likelihood of earthquake insurance uptake include the type of housing (renting vs. owning), level of education, and annual income.

In addition to these factors, respondents' attitudes toward earthquakes were also found to be significant factors that influence earthquake insurance uptake. Specifically, individuals who had experienced moderate earthquake damage or expressed concerns about earthquake safety, as well as those who believed that the state should regulate environmental quality, were more likely to insure against earthquakes. Finally, working in the oil and gas industry, receiving oil and gas revenue leases, and having multiple oil and gas companies on their property were also found to significantly influence the decision to acquire earthquake insurance.

### Predictive modeling results of earthquake insurance uptake

Before we present prediction results of which individuals were likely to acquire earthquake, we first report performance measures of the two tree-based ML algorithms. The performance measures indicate that a decision tree model had an accuracy rate of 89.55%, while the random forest classifier achieved a perfect classification rate of 100%. These findings imply that a decision tree was able to correctly predict about 90% of the time those that would insure against earthquakes as well as those that did not insure against earthquakes while the random forest did so by 100%. When it comes to precision, we found that a decision tree classifier achieved a precision score of 83.58% implying that out of the total of those that have earthquake insurance, the decision tree classifier was able to correctly predict 83.58% of them to have earthquake insurance. Interestingly, the random forest classifier correctly predicted all positive cases, achieving a perfect precision score of 100%.

In terms of sensitivity, the decision tree and random forest classifiers achieved sensitivity scores of 28.33% and 100%, respectively. In terms of specificity, which represents the proportion of observations without earthquake insurance that are correctly identified out of the total number of uninsured observations, the decision tree classifier exhibited a specificity score of 99%, while the random forest classifier achieved a perfect score of 100%. Overall, our findings suggest that a random forest classifier performed better than a decision tree on these predictions, a result that parallels Geetha et al. ^57^. This could be because random forests create an ensemble of decision trees and average their predictions which helps reduce variance and cancel out individual decision tree errors^40^.

**Figure 3:**
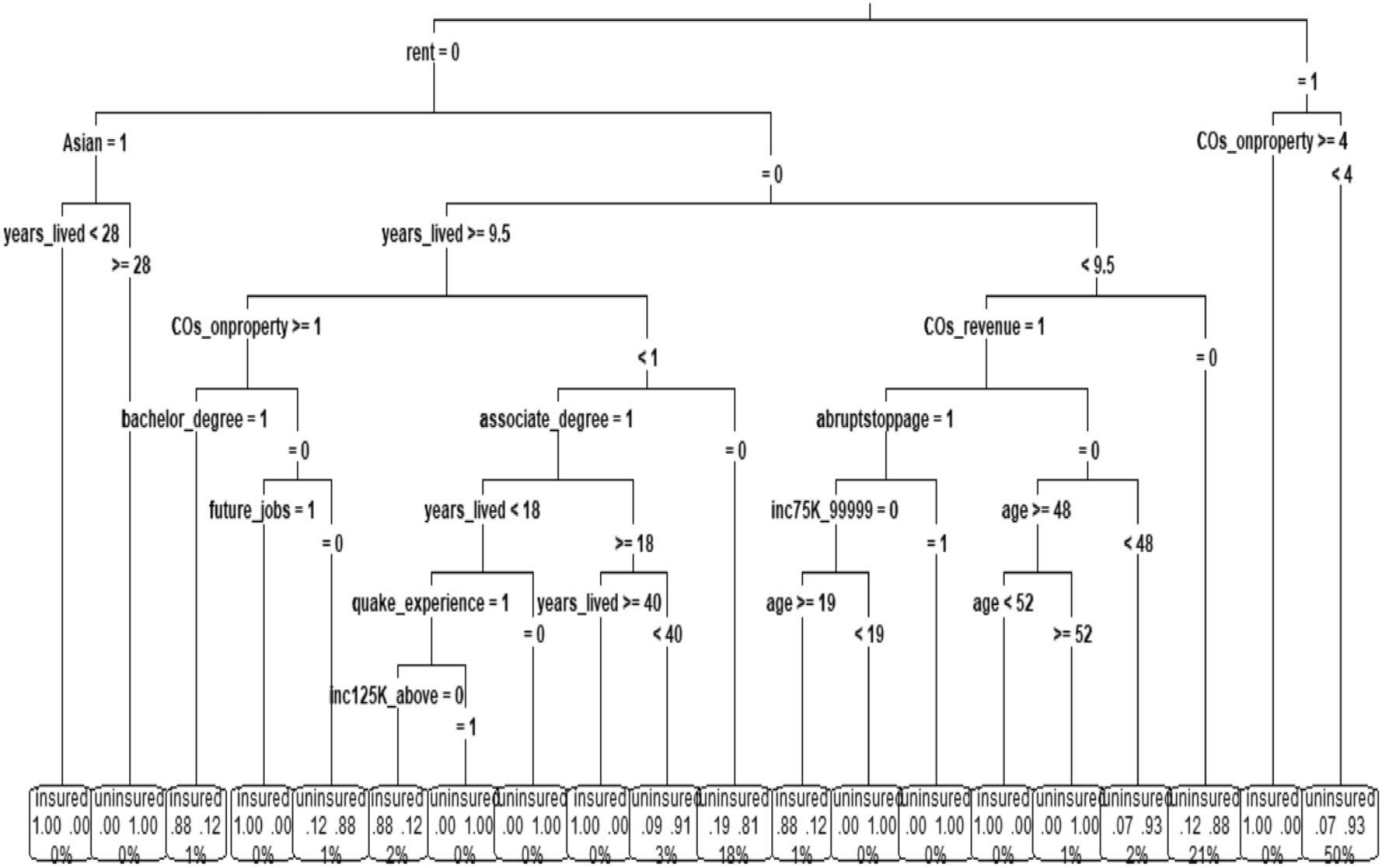
Decision tree.

With regard to predicting which individuals are likely to acquire earthquake insurance, a decision tree was generated and depicted in Fig. 3. This decision tree consists of a root node, nine levels, 20 leaf nodes, and 38 decision nodes. The importance of the predictors in the decision tree is based on the information gained from each variable^58^. In our decision tree, the internal/decision nodes represent the variables that contribute the most to predicting which individuals would take earthquake insurance. On the other hand, the leaf nodes represent the outcome variable, which indicates whether a respondent takes earthquake insurance or not. Notably, our decision tree shows that whether a resident rents their property or not is the most important predictor of earthquake insurance uptake, as shown in the primary branch of the tree.

Our decision tree results unveil intricate patterns that shed light on which individuals based on the covariates would acquire earthquake insurance. For individuals who rent their property and have fewer than four oil and gas companies operating on their premises, the estimated probability of insuring their property against earthquakes is a mere 7%. This result may be due to renters possibly assuming that the property owner's insurance covers earthquake damage, leading to a lower likelihood of seeking additional coverage. However, a stark contrast emerges when individuals contend with at least four oil and gas companies on their property, where the likelihood of insuring their property against earthquakes surges to a staggering 100%. One plausible explanation could be that the presence of numerous companies reflects higher risk perception, motivating individuals to secure earthquake insurance. This aligns with the idea that proximity to oil and gas activities may raise concerns about induced seismicity and, consequently, the need for coverage^11^.

Conversely, a different narrative emerges for individuals who do not rent a property, are not of Asian ethnicity, have lived in Oklahoma for less than 9.5 years, and receive gas leases from oil and gas companies, yet do not believe that wastewater injection should be stopped abruptly. In this scenario, such individual’s predicted probability of insuring their property against earthquakes plummets to 0%. The absence of insurance interest within this group might relate to a perception of low earthquake risk, potentially influenced by their shorter duration of residence and trust in wastewater injection practices. Conversely, a distinct subgroup of individuals, characterized by non-renters who are not of Asian ethnicity, have lived in Oklahoma for less than 9.5 years, receive gas leases from oil and gas companies, advocate for the cessation of wastewater injection, do not have an annual income between $75,000 and $99,999, and are older than 19 years, emerge as the most likely to secure earthquake insurance, with an elevated 88% probability. The high likelihood among this subgroup could be attributed to their greater awareness of earthquake risks due to their opposition to wastewater injection and possibly lower income levels, which could make them more risk-averse. Intriguingly, this subgroup constitutes merely 1% of the observations within the training dataset. We find many results from the decision tree and to conserve space, the remaining pathways and individuals’ associated predicted probabilities of earthquake insurance uptake are outlined in Table 4.

**Table 4:** The 19 rules extracted from the decision tree.

Rule No	The Rule
Rule 1	The estimated probability of insuring a property against earthquakes is 7% for individuals who rent a property and have less than 4 oil and gas companies on the property. This subgroup represents 50% of the observations in the training dataset
Rule 2	For individuals who rent a property and have more than four oil and gas companies on the property, the estimated probability of insuring the property against earthquakes is 100%. This subgroup represents 0% of the observations in the dataset
Rule 3	For individuals who do not rent a property, are not of Asian ethnicity, have lived in Oklahoma for less than 9.5 years, do not receive gas leases from oil and gas companies, the estimated probability of insuring the property against earthquakes is 12%. This subgroup represents 21% of the observations in the training dataset
Rule 4	For individuals who do not rent a property, are not of Asian ethnicity, have lived in Oklahoma for less than 9.5 years, and receive gas leases from oil and gas companies, do not believe wastewater injection should be stopped abruptly, and are younger than 48 years old, the estimated probability of insuring the property against earthquakes is 7%. This subgroup represents 2% of the observations in the training dataset
Rule 5	For individuals who do not rent a property, are not of Asian ethnicity, have lived in Oklahoma for less than 9.5 years, and receive gas leases from oil and gas companies, do not believe wastewater injection should be stopped abruptly, and are older than 52 years old, the estimated probability of insuring the property against earthquakes is 0%. This subgroup represents 1% of the observations in the training dataset
Rule 6	For individuals who do not rent a property, are not of Asian ethnicity, have lived in Oklahoma for less than 9.5 years, and receive gas leases from oil and gas companies, do not believe wastewater injection should be stopped abruptly, and between 48 and 52 years old, the estimated probability of insuring the property against earthquakes is 100%. This subgroup represents 0% of the observations in the training dataset
Rule 7	For individuals who do not rent a property, are not of Asian ethnicity, have lived in Oklahoma for less than 9.5 years, and receive gas leases from oil and gas companies, believe wastewater injection should be stopped abruptly, have annual income between $75,000 and $99,999, the estimated probability of insuring the property against earthquakes is 0%. This subgroup represents 0% of the observations in the training dataset
Rule 8	For individuals who do not rent a property, are not of Asian ethnicity, have lived in Oklahoma for less than 9.5 years, and receive gas leases from oil and gas companies, believe wastewater injection should be stopped abruptly, do not have annual income between $75,000 and $99,999, and are younger than 19 years old, the estimated probability of insuring the property against earthquakes is 0%. This subgroup represents 0% of the observations in the training dataset
Rule 9	For individuals who do not rent a property, are not of Asian ethnicity, have lived in Oklahoma for less than 9.5 years, and receive gas leases from oil and gas companies, believe wastewater injection should be stopped abruptly, do not have annual income between $75,000 and $99,999, and are older than 19 years old, the estimated probability of insuring the property against earthquakes is 88%. This subgroup represents 1% of the observations in the training dataset
Rule 10	Individuals who do not rent their property, are not of Asian ethnicity, have lived in Oklahoma for over 9.5 years, have less than one oil and gas company on their property, and do not possess an associate degree, are estimated to have a 19% probability of insuring their property against earthquakes. This subset represents 18% of the observations in the training dataset
Rule 11	Individuals who do not rent their property, are not of Asian ethnicity, have lived in Oklahoma between 18 and 40 years, have less than one oil and gas company on their property, and have an associate degree, are estimated to have a 9% probability of insuring their property against earthquakes. This subset represents 3% of the observations in the training dataset
Rule 12	Individuals who do not rent their property, are not of Asian ethnicity, have less than one oil and gas company on their property, have lived in Oklahoma for over 40 years, and have an associate degree, are estimated to have a 100% probability of insuring their property against earthquakes. This subset represents 0% of the observations in the training dataset
Rule 13	Individuals who do not rent their property, are not of Asian ethnicity, have lived in Oklahoma for less than 18 years, have less than one oil and gas company on their property, have an associate degree, and do not have past earthquake experience, are estimated to have a 0% probability of insuring their property against earthquakes. This subset represents 0% of the observations in the training dataset
Rule 14	Individuals who do not rent their property, are not of Asian ethnicity, have lived in Oklahoma for less than 18 years, have less than one oil and gas company on their property, have an associate degree, have past earthquake experience, and have annual income of at least $125,000, are estimated to have a 0% probability of insuring their property against earthquakes. This subset represents 0% of the observations in the training dataset
Rule 15	Individuals who do not rent their property, are not of Asian ethnicity, have lived in Oklahoma for less than 18 years, have less than one oil and gas company on their property, have an associate degree, have past earthquake experience, and do not have annual income of at least $125,000, are estimated to have an 88% probability of insuring their property against earthquakes. This subset represents 2% of the observations in the training dataset
Rule 16	Individuals who do not rent their property, are not of Asian ethnicity, have lived in Oklahoma for at least 9.5 years, have at least one oil and gas company on their property, do not have an undergraduate degree, and do not expect to be hired by oil and gas companies in the future, are estimated to have a 12% probability of insuring their property against earthquakes. This subset represents 1% of the observations in the training dataset
Rule 17	Individuals who do not rent their property, are not of Asian ethnicity, have lived in Oklahoma for at least 9.5 years, have at least one oil and gas company on their property, do not have an undergraduate degree, and expect to be hired by oil and gas companies in the future, are estimated to have a 100% probability of insuring their property against earthquakes. This subset represents 0% of the observations in the training dataset
Rule 18	Individuals who do not rent their property, are not of Asian ethnicity, have lived in Oklahoma for at least 9.5 years, have at least one oil and gas company on their property, and have an undergraduate degree are estimated to have an 88% probability of insuring their property against earthquakes. This subset represents 1% of the observations in our dataset
Rule 19	Individuals who do not rent their property, are of Asian ethnicity, have lived in Oklahoma for at least 28 years, are estimated to have a 0% probability of insuring their property against earthquakes. This subset represents 0% of the observations in the training dataset
Rule 20	Individuals who do not rent their property, are of Asian ethnicity, have lived in Oklahoma for less than 28 years, are estimated to have a 100% probability of insuring their property against earthquakes. This subset represents 0% of the observations in the training dataset

**Figure 4:**
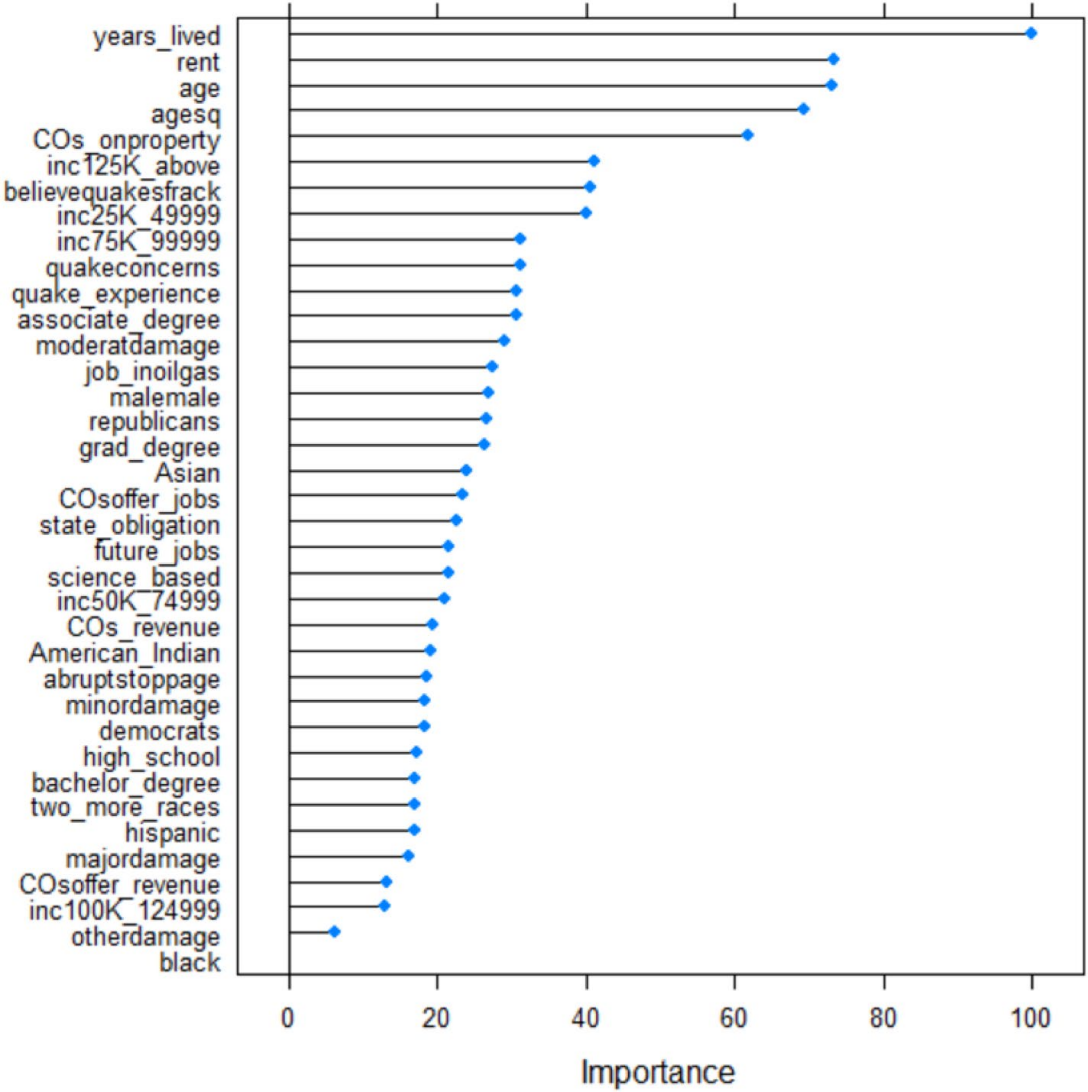
Variable Importance from a random forest algorithm.

In Fig. 4, the order of importance of variables in forecasting which individuals are inclined to obtain earthquake insurance, in accordance with the random forest classifier, is presented. In Fig. 4, we present the order of importance of variables in predicting earthquake insurance uptake. Our analysis reveals that the length of an individual's residence in Oklahoma is the single most influential predictor of who has earthquake insurance. Following this, property rental status emerges as the next vital factor. Moreover, individual age and the presence of oil and gas companies operating on one's property also exhibit significant contributions to the likelihood of securing earthquake insurance. In summary, the duration of residency in Oklahoma, property rental status, age, and the extent of oil and gas company activities on their property are all prominent, contributing by at least 50% towards the prediction of earthquake insurance acquisition. Additionally, numerous other variables play a substantial role in forecasting who is likely to obtain earthquake insurance. Conversely, the variable related to respondents identifying as Black or African American is found to make the smallest contribution to the prediction of earthquake insurance uptake, indicating its limited relevance in this context.

## Discussion

The recent surge in natural disasters worldwide, including the increasing occurrence, intensity, and severity of earthquakes, highlights the need for effective risk management tools, such as insurance packages, to ensure the safety of nations and their citizens^1,3^. The widespread impact of these catastrophic events, affecting many people simultaneously^47^, emphasizes the importance of understanding people's preparedness for earthquakes and their decisions to opt for earthquake damage insurance. Our study employs a multifaceted approach, integrating logit, ridge regression, and Least Absolute Shrinkage and Selection Operator (LASSO) classifiers to identify influential factors of earthquake insurance uptake. Additionally, we took advantage of the predictive power of decision tree and random forest classifiers to identify individuals likely to invest in earthquake insurance based on relevant covariates. Our analyses yield valuable insights for policymakers, facilitating the development of more robust insurance strategies aimed at safeguarding individuals and communities from the profound repercussions of seismic events.

The logit, ridge regression, and LASSO demonstrated their ability to provide consistent results on the influential factors that affect earthquake insurance uptake. This was evidenced by the LASSO not shrinking away any factor coefficients with coefficient signs and magnitudes being close to those from the logit and ridge classifiers. On the other hand, decision trees and random forest classifiers generated robust findings by accurately predicting which individuals are likely to acquire earthquake insurance based on the relationships within the data^59^. The random forest classifier had a 100% score across accuracy, precision, sensitivity, and specificity with the decision tree coming close with regard to accuracy and specificity.

Our findings on influential factors of earthquake insurance uptake suggest that age is a significant factor in earthquake insurance uptake, with younger residents being less likely to insure against earthquake damage initially but becoming increasingly more likely to do so as they age. This trend may be due to older individuals becoming more aware of the need for insurance as they experience changes in lifestyle and gain a greater understanding of the potential danger posed by earthquakes. The influence of lifestyle on insurance adoption is an important consideration, as it can affect the perceived value of insurance among individuals. For instance, Sidi et al.^60^ have noted that the demand and supply of insurance services related to flooding are affected by various factors, including age, economic circumstances, family status, and lifestyle. Similarly, the findings about the Hurricane Katrina disaster suggest that age is an important factor in the decision to purchase flood insurance, with older residents being more likely to insure their properties against floods compared to younger residents^61^. Thus, policymakers should consider these age-related factors when developing strategies to promote earthquake insurance uptake, particularly targeting younger residents who may be less likely to prioritize insurance.

Surprisingly, the results of our study indicate that male respondents are more likely to have earthquake insurance than female respondents. We had initially hypothesized that women would be more cautious of environmental risks and therefore more likely to have earthquake insurance^11,41^. However, it is possible that insurance policies are often registered in the name of the male household head, which could explain this finding. In terms of ethnicity and race, our results align with our expectations: respondents of Hispanic origin are more likely to have earthquake insurance, while Asian respondents and those of two or more races are also more likely to insure against earthquakes than White respondents. This finding is consistent with Ansolabehere and Konisky^42^, who found that minority groups are more skeptical and concerned about such environmental disasters. It is possible that earthquakes linked to wastewater injection motivate them to have earthquake damage insurance in case of an unexpected seismicity.

Our results indicate that Democrats are less likely to own earthquake insurance than Independents, contrary to what was expected. This finding is surprising given that previous studies have shown that Democrats generally support wastewater injection regulation, which has been linked to increased seismic activity in Oklahoma^11^. Wines^62^ noted that Democratic state lawmaker, Cory Williams, advocated for more robust action on the quakes and criticized the state's response to the surge in earthquakes. Furthermore, the study found that respondents who have lived in Oklahoma for a longer period are more likely to have earthquake damage insurance. This result is reasonable because those who have lived in Oklahoma longer would have experienced more earthquakes, making them more likely to have their property and residence insured.

Our study also found that renters are less likely than homeowners to have earthquake damage insurance. This is expected since renters may be less interested in seeking earthquake insurance as they can easily move away. In terms of respondents' education levels, those with higher education levels (e.g., associate, undergraduate, or graduate degrees) are more likely to have earthquake damage insurance than those with lower education levels. This could be because people with higher education levels are more aware of the benefits of insurance and the risks associated with earthquake damage.

Respondents with higher incomes (at least $25,000 annual gross income) are more likely to have earthquake insurance than those with lower incomes. This finding is consistent with Browne et al.'s^61^ observation of a strong association between wealth and insurance demand, as well as a preference for insuring against high likelihood, low level consequence risks. People with relatively higher incomes may have more assets and property to protect, making them more likely to invest in earthquake insurance to recover from potential losses.

We found that respondents whose property or residence experienced moderate earthquake damage or other earthquake damage are more likely to have earthquake damage insurance compared to those whose property did not suffer any damage from earthquakes. This result is consistent with Choi et al.’s ^63^. findings on risk preparedness for tornadoes: individuals who have experienced the devastating effects of natural disasters would be more likely to seek protection through disaster insurance. A study conducted by Ivčević et al.^64^ in Italy found that locals were willing to invest in mitigating climate change-related threats, which supports the idea that individuals are willing to take measures to protect themselves from natural disasters. In a similar vein, Osberghaus^65^ and Hong^66^ suggested that individuals tend to take preventive measures against flood damage when they have experienced historical damage and anticipate future damage. Furthermore, respondents who were concerned about earthquakes are more likely to insure against earthquake damage than those who were not. This finding is consistent with the observations of Pothon et al.^67^ that individuals are more likely to insure their homes or property against earthquakes if they believe that a devastating earthquake is imminent and are concerned about potential damage. Overall, these findings suggest that personal experiences and attitudes towards natural disasters play a crucial role in the adoption of disaster insurance.

Moreover, we found that respondents who believe the state has a responsibility to regulate the quality of the environment and the actions of fracking companies are significantly more likely to have earthquake insurance. However, we found that financial assistance provided by the government after natural disasters may be perceived as premium-free and influence demand for natural disaster insurance, which is in line with the work of Browne and Hoyt^68^ on aid hazards. Browne and Hoyt^68^ found that individuals at risk may not purchase insurance due to their expectation of receiving aid from various sources.

Furthermore, we found that an increase in the number of oil and gas drilling companies at respondents' residences and respondents working in the oil and gas industry is positively associated with higher odds of having earthquake insurance. This finding suggests that individuals who have experience with oil and gas companies may have a greater awareness of the potential risks associated with fracking and may be motivated to mitigate the risk of earthquake damage by purchasing insurance. In contrast to flood insurance, where proximity to flood-prone areas may not affect an individual's decision to purchase insurance^69^, the presence of oil and gas companies on a property exposes it to induced earthquakes related to wastewater injection, potentially increasing the likelihood of individuals purchasing earthquake insurance.

Our findings on the effect of individuals’ location, experience, and risk perception on insurance uptake are consistent with previous studies. For example, Antwi-Boasiako's^29^ study in Ghana found that homeowners with internal locus control of location are more likely to insure against natural disasters. Similarly, Osberghaus^65^ observed in Germany that homeowners’ likelihood of insuring against flood damage increases with historical damage and the prospect of future damage. Yu et al.^70^ found that factors such as households' risk perception, education level, and profession significantly affect the intention to adapt to earthquake risks in rural China.

In addition, risk tolerance is a crucial factor that influences the uptake of disaster insurance among homeowners. Landry and Turner^71^ on the Georgia Coast found that risk tolerance, attitude towards community risk management initiatives, wealth exposure, and perceived damages all determine the adoption of disaster insurance. Athavale and Avila^44^ suggest that the demand for earthquake insurance increases with the level of risk, indicating that homeowners take earthquake insurance due to the potential risks associated with earthquakes.

With regards to predicting which individuals are more likely to acquire earthquake insurance, most results from the decision tree are consistent with factors identified by a random forest classifier that produced perfect predictions and ranked variables by their importance in influencing earthquake insurance uptake. Our decision tree results showed that individuals who rent property and have a limited number of oil and gas companies operating on their premises demonstrate a relatively low likelihood (7%) of insuring their property. However, this subgroup constitutes a significant portion of the dataset, accounting for 50% of the observations. In contrast, individuals with the same renting status but with a higher number of oil and gas companies on their property exhibit a much higher probability (100%) of obtaining earthquake insurance, despite comprising a smaller percentage of the dataset (0%). This implies that the presence of these companies significantly influences insurance decisions, a variable that the random forest classifier also indicated to be important in predicting who acquires earthquake insurance.

Moreover, individuals who do not stay on rented property, have resided in Oklahoma for less than 9.5 years, and do not receive gas leases from oil and gas companies have a 12% probability of insuring their property, with a considerable representation of 21% in the dataset. Age plays a crucial role within this subgroup. Those under 48 years old display a 7% probability, while those older than 52 years old are much less likely (0%) to insure their property. Interestingly, individuals between 48 and 52 years old in this group are highly inclined to obtain earthquake insurance (100%). Another significant factor that influences insurance decisions is the perception of wastewater injection practices. For individuals with characteristics similar to the aforementioned group but who do not believe wastewater injection should be abruptly halted and have an annual income between $75,000 and $99,999, the probability of insuring their property is 0%. In contrast, individuals with the same characteristics but who are older than 19 years old are much more likely (88%) to acquire earthquake insurance.

The findings continue to underscore the complexity of insurance decision-making. Variables such as education level and duration of Oklahoma residency also come into play. Notably, individuals who have lived in Oklahoma for over 9.5 years, hold an associate degree, and do not anticipate working for oil and gas companies have a 12% probability of insuring their property, while those with similar characteristics but with job expectations in the oil and gas industry have a 100% probability.

These findings are consistent with prior research in behavioral economics and insurance decision-making. Studies such as Doherty and Schlesinger^72^ and Outreville^73^ emphasized the role of risk perceptions and external factors in insurance choices. Moreover, in the context of environmental risk perceptions, Slovic^74^ highlighted the impact of public attitudes and beliefs on risk-related decisions. The results from this study further underscore the significance of individual beliefs, experiences, and contextual factors in earthquake insurance uptake.

Overall, the findings presented in this study could inform future policy decisions aimed at mitigating the impact of natural and induced hazards in Oklahoma and other regions facing similar risks. Collectively, our study and others (e.g. ^64,65^,) highlight the importance of understanding individuals' risk perception, knowledge, and experience with natural disasters when examining their decision to purchase disaster insurance.

## Conclusion

The present study sought to contribute to the design and evaluation of effective earthquake insurance compensation mechanisms by providing valuable insights to policymakers, insurance companies, and individuals. We used supervised machine learning techniques to identify influential factors of earthquake insurance uptake as well as predict individuals that would take earthquake insurance. Our data were collected from residents from Oklahoma, USA.

Based on our study's findings, we draw the following conclusions and policy implications. First, the survey results showed that only 14.4% of the total sample had earthquake damage insurance in Oklahoma, which is cause for concern given the increased likelihood of earthquakes and seismic activity in the state^38^. This low percentage of insurance coverage highlights the need for increased awareness and education campaigns aimed at encouraging residents in Oklahoma and other earthquake-prone regions around the world with little or no earthquake insurance^75^, to encourage purchase of earthquake damage insurance. This will help to ensure that residents can protect their property and livelihoods in the event of catastrophic losses from earthquakes. According to the Organization for Economic Co-operation and Development (OECD)^3^, raising awareness about the importance of financial preparedness may include communicating about the expected allocation of disaster costs, particularly who is responsible for those costs, as well as providing information about the availability and key characteristics of disaster risk financing tools. It is therefore recommended that policymakers and stakeholders take proactive measures to increase awareness of the importance of earthquake damage insurance and work to create policies that encourage more residents to obtain insurance coverage.

Second, the empirical findings derived from logit, ridge regression, and LASSO, focusing on the determinants of earthquake insurance adoption, are consistent with the results obtained from decision tree and random forest classifiers. However, it's worth noting that the decision tree analysis goes a step further by delving into intricate nonlinear relationships, a dimension that logit, ridge, and LASSO are inherently limited to exploring. Essentially, we found that many socio-demographic factors affect people's decisions to insure against earthquakes. Women and younger respondents were less likely to have earthquake insurance, underscoring the need for targeted awareness campaigns aimed at these groups. Additionally, respondents of Hispanic or Asian descent and those identifying as multiracial were more likely to have earthquake insurance than White respondents. it is therefore important to encourage earthquake-prone residents of all races and ethnicities to insure against earthquakes, not just targeting awareness programs to minority racial ethnic groups.

Third, our study emphasized the role of education, as respondents with higher levels of formal education were more likely to have earthquake insurance showing the potential importance of knowledge and decision analysis skills in making life choices such as earthquake insurance uptake^76^. We note that higher-income groups are more likely to insure against earthquakes due to the value of their assets.

Fourth, there are several variables related to respondents’ attitudes toward earthquakes that significantly explain people’s decision to have earthquake insurance. Respondents who have experienced moderate property damage or consider earthquakes a safety issue consider earthquake insurance and the regulation of wastewater injection as important measures that could mitigate earthquake risks and minimize mental concerns going forward. Additionally, the finding that respondents connected to the fracking industry are still interested in purchasing earthquake insurance highlights the importance of an efficient insurance system that compensates earthquake victims. Therefore, our results suggest that state regulation of activities linked to earthquakes and overseeing an effective insurance system should be a priority for policymakers to address the risks of earthquakes in Oklahoma and elsewhere.

Moreover, our analysis demonstrates that both decision trees and random forests offer robust predictive capabilities for identifying individuals who would and would not acquire earthquake insurance. The decision tree achieved an accuracy rate of approximately 90% in correctly classifying these groups, while the random forest excelled with an impressive accuracy rate of 100%. Delving into precision, the decision tree classifier exhibited a commendable score of 83.58%, signifying its ability to accurately predict 83.58% of those with earthquake insurance. Intriguingly, the random forest classifier achieved a remarkable perfect precision score of 100%. While both methods are effective, the random forest's exceptional precision and robustness make it an encouraging choice for modeling earthquake insurance uptake and other classification problems, offering heightened confidence in its predictions.

## Data Availability

The datasets generated during and/or analyzed during the current study are not publicly available due to privacy/ethical reasons but are available from the corresponding author on reasonable request.
